# #GoingtotheFair: a social media listening analysis of agricultural fairs^1^

**DOI:** 10.1093/tas/txaa139

**Published:** 2020-07-20

**Authors:** Julie A Mahoney, Nicole J O Widmar, Courtney L Bir

**Affiliations:** 1 United Animal Health, Sheridan, IN; 2 Department of Agricultural Economics, Purdue University, West Lafayette, IN; 3 Department of Agricultural Economics, Oklahoma State University, Stillwater, OK

**Keywords:** agricultural fair, fairs, livestock, online media sentiment, zoonotic disease

## Abstract

Agricultural fairs provide one of the last frontiers, and largest stages, for showcasing livestock agriculture to the public. However, public funding, attendance revenue, animal biosecurity, and public health concerns are all aspects worthy of conversation and increased research attention given the interaction between livestock animals and the general public in fair and festival settings. A prominent social media listening and data analytics platform was used to quantify online and social media chatter concerning agricultural fairs during a 27-mo period. A general search for online media referencing agricultural fair keywords was designed; social and online media mentions of agricultural fairs (*n* = 2,091,350 mentions) were further queried according to their reference to livestock, fair food, or the major agricultural product producing species of dairy and beef cattle (*n* = 68,900), poultry (*n* = 39,600), and swine (*n* = 31,250). Numbers of search results were found to be seasonal and Twitter was the single largest domain for all fair-related results; in contrast, the majority of livestock-related media was generated by news sources rather than from Twitter. On a weekly basis, the percentage of fair livestock mentions with species-specific reference was highly variable ranging from 0% to 86.8% for cattle, 0% to 85.7% for poultry, and 0% to 76.9% for swine. In addition to quantifying total search hits or mentions, the positivity/negativity of the search results was analyzed using natural language processing capabilities. The net sentiment quantified is the total percentage of positive posts minus the percentage of negative posts, which results in a necessarily bounded net sentiment between −100% and +100%. Overall net sentiment associated with mentions of agricultural fairs was positive; the topics garnering the highest positive sentiments were fair food and cattle (both 98% positive). Online discussion pertaining to agricultural fairs and swine was overall positive despite references to swine flu outbreaks. In conclusion, livestock and animal products had positive net sentiment over the time period studied, but there are multiple aspects of agricultural fairs worthy of further investigation and continued vigilance, including zoonotic disease risk and public perceptions of livestock industries.

## INTRODUCTION

Attention in recent years has been placed on the development of agritourism venues to connect the general public to agricultural industries and food production (Phillip et al., 2010), yet agricultural fairs and festivals have long contributed to the connection between agrarian and nonagrarian lifestyles. Agricultural fairs uniquely provide social engagement, entertainment, youth development opportunities, and exhibition of a variety of agricultural product sectors. Fairs also serve an educational purpose, with exhibits showcasing new technologies and/or stakeholder groups providing outreach to nonagricultural visitors. The longstanding tradition of the agricultural fair is widespread both geographically and financially, with practical support from many stakeholder groups as evidenced by food animal producer group sponsorships and involvement (i.e., pork tent, beef tent, and other livestock producer group events or booths).

Despite their cultural legacy, agricultural fairs have not been immune to scrutiny for associated zoonotic disease transmission and biosecurity risks (LeJeune and Davis, 2004; Thunes and Carpenter, 2007; Bowman et al., 2017; CDC, 2018). In recent years, biosecurity and biocontainment concerns regarding foreign animal disease and highly pathogenic avian influenza prompted cautionary measures of canceling long-established exhibitions and shows (DeVita and Reimink, 2015; NPPC, 2019). Since 2012, the U.S. Department of Health (USDA) One Health initiative has devoted resources to address the zoonotic health risks faced by animals and humans alike (USDA—APHIS, 2019) with specific research and educational facets, including avian influenza and swine influenza serotypes H1N1, H1N2, and H3N2. These diseases present a discernable public health liability whenever commingling of people and agricultural livestock occurs, an occasion for which information on public opinion and attitudes could prove useful. However, literature is sparse regarding the assessment of the fairgoer’s awareness of zoonotic disease transmission risk.

Scant data also exist on how agricultural fairs affect the public’s perception of various agricultural endeavors, especially livestock enterprises. Agritourism enterprises, farmer’s markets featuring animal products (meats, cheese, and dairy), farm tours for school children, and other endeavors have all received increasing attention from animal agriculture industries in recent years as industry groups have sought to better connect with end consumers of livestock products. Cummins et al. (2016) highlighted a need to better understand the demographics and perceptions of animal agritourism visitors to better discern their demands for production systems and product attributes. Additionally, the raising and exhibition of livestock are also facing public scrutiny. Byrd et al. (2017) asked a representative sample of U.S. residents about the acceptance of the use of animals for various uses, ranging from the largest acceptance for food production (93% acceptance of egg production; 92% acceptance of milk production) to using animals in circuses (only 52% of respondents found circus animals acceptable). Eighty-three percent of the 825 respondents found the use of animals for livestock shows (i.e., state or county fairs) acceptable, making it slightly higher in acceptance than petting zoos (79%) but notably lower than raising animals for meat (88%; Byrd et al., 2017).

For some time, the social sciences have employed data analytics tools to study online social media and internet-based news. Although adoption has been slower among the biological sciences, there has been a growing emphasis on the application of big data analytics in the animal agriculture industry (Morota et al., 2018). Social scientists have become increasingly interested in self-proclaimed text format data, including that from social listening (Carr et al., 2015), Google search data (Stephens-Davidowitz 2018), and news media data collected online (Tonsor and Olynk 2010). Similar to tools enabling electronic research-oriented searches, such as those offered by LexisNexis (New York, NY), the NetBase software platform (NetBase Solutions, Inc.; Mountain View, CA) makes possible large-scale internet searches of social media, blogs, reviews, websites, and media.

This analysis sought to quantify and analyze data scraped from social media to gain understanding of the discussions, posts, and media prevalence about agricultural fairs on the Web. Information pertaining to online media, including the volume generated when that media is generated, and by whom, are all of interest to industry stakeholders invested in agricultural fairs. The objectives of this analysis include utilizing large data processing software to quantify and interpret online accessible media and conduct social media sentiment analysis to discern the negativity or positivity associated with online fair-related news and online media. Specifically, it is hypothesized that a measurable and socially significant portion of the media surrounding fairs is related to animal agriculture, as opposed to carnival aspects of the fair. Furthermore, it is hypothesized that considerable attention is placed on fair foods, which are often affiliated with animal agriculture groups, such as a state’s pork or beef association group. Understanding the online media to glean insights into what the public sees in the fair is expected to contribute to a greater quantification of public perception of animal agriculture via one of the most public-facing events of the year wherein livestock agriculture and the general public interact, namely the fair.

## MATERIALS AND METHODS

To quantify the number of internet posts containing keywords related to agricultural fairs, food, and livestock, the NetBase platform was used to search and analyze posts from July 1, 2017 to September 15, 2019. This 27-mo timeframe was chosen to detect online and social media patterns across successive years particularly during the late summer months (July, August, and September) when most state fair exhibitions occur (see Supplementary Data for an author-amassed 2017–2019 dates of prominent agricultural state-level fairs). The search parameters were finalized, queried, and summary statistics and aggregated findings downloaded on September 26, 2019. Since social media posts and media posted online are subject to removal and/or reinstatement at the discretion of their author or domain, the results of the formal data collection exercise must be considered a snapshot in time of fluid social media communication.

To identify and quantify social media posts and mentions related to agricultural fairs, a query was parameterized using 12 search keywords. These 12 search keywords (documented in parenthesis as follows) were both general (“agricultural fair” and “going to the fair”) and specific to fairs of various scale and type (“state fair,” “county fair,” “parish fair,” and “youth fair”), and included corresponding hashtags (#agriculturalfair, #agfair, #statefair, #countyfair, #youthfair, and #goingtothefair). Targeting specific phrases or hashtags is important especially because Twitter postings (“tweets”) have limited characters and consequently are short in length (Fried et al., 2014). However, keywords were designed to return social media hits and conversations generically referencing agricultural fairs while remaining nonselective for specific conversations with a zoonotic disease or youth development program focus in order to allow these discussion topics to surface organically if they truly commanded a notable percentage of social media attention. Since the primary terms also returned conversations concerning political and industrial topics, five terms were employed to exclude conversations referring to “free & fair election,” “free, FAIR elections,” “free, fair and credible,” “free, fair and open internet,” and “fair trade.” The NetBase platform is capable of searches across global geographical coordinates, so, in the present analysis, the query was restricted to geolocations within the continental United States. Likewise, while searches in multiple languages are possible, the level of fluency required to interpret phrases, and especially slang or cultural context, limit interpretability. Thus, this search and related analysis were conducted exclusively for media and posts in the English language.

**Figure 1.**
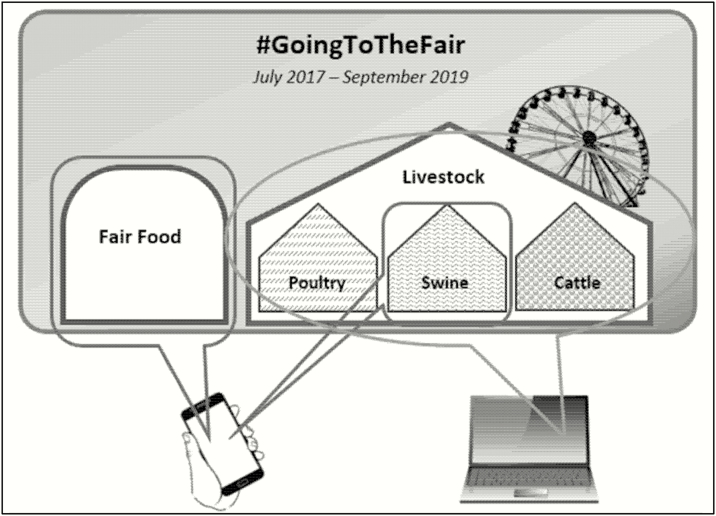
Social media conversations referencing agricultural fairs were queried to further characterize how the conversations of the present digital age incorporate discussion of livestock species and fair food.

Agricultural fair posts/mentions returned by the original query were further filtered into subsets of conversation by topic (Fig. 1). To identify those that included a reference to food, 230 terms were chosen as subsearch parameter keywords. These search words and hashtags were chosen to represent various fair food items across a wide geographic region (the continental United States) and included several meat-specific terms, including pork and beef Checkoff promotional slogans, as well as Instagram tags and popular food terms identified in other research (see Supplementary Data for complete listing). Hashtags such as #dinner, #meal, #snack, and #lunch have been successfully used to query data (Fried et al., 2014), while tags such as #food, #foodporn, #instafood, and #instagood are among the most popular social media hashtags concerning food (Mejova et al., 2016; Rich et al., 2016). Some terms (i.e., “beef,” “chicken,” and “turkey”) can be interchangeably used in reference to both the live animal and the respective food product; thus, it is possible that these food search keywords could have returned mentions related to live animals in addition to food items.

Similarly, the aggregate of agricultural fair posts/mentions was filtered to identify a subset of fair conversations that referenced livestock based on 219 keywords. These words and hashtags included terms that were generically related to animals, that is, barn, manure, and rodeo; terms encompassing several species, including horses, sheep, goats, and rabbits; and popular Instagram tags for several common species. To further identify social media conversations specific to the major agricultural product-producing species (poultry, dairy cattle, beef cattle, and swine), species filter terms were a narrowed subset of the livestock filtering terms. The 44 terms and hashtags specific to beef and dairy livestock industries were consolidated into a single search category (“cattle”) due to ubiquitous terms, such as “cow” and “calf,” as well as a possible lack of consumer knowledge to accurately differentiate between dairy and beef cattle terms. Poultry-related fair conversations were filtered using 69 terms and hashtags related to chickens, turkeys, and ducks. Swine-related fair conversations were filtered using 35 terms and hashtags related to pigs.

### Social Media Sentiment Analysis

Sentiment analysis attempts to detect and quantify the overall sentiment that is available online and in social media to understand collective views or positive/negative propensity toward a topic as a whole. Sentiment analysis using social media-derived data fundamentally transforms qualitative information into a numeric score (Thelwall et al., 2011). Sentiment quantification can be used to assess attitudes toward brands on Twitter (Mostafa, 2013) or in the context of developing consumer products (Carr et al., 2015). The uses of social media net sentiment are wide and varied, yet largely untapped for studying agricultural industries and related topics. Social media listening or web-scraped data is becoming established with literature and computer sciences devoted to algorithm development over the past decade and ongoing. However, how to best work with the large textual data sets that are generated remains debated in many academic fields.

A sentiment score, which is a numeric value, was assigned using Netbase’s patented Natural Language Processing (NLP) engine, which analyzes sentiment for every subject in a sentence (Netbase, 2018). The net sentiment referenced throughout this analysis is the total percentage of positive posts minus the percentage of negative posts, which results in a necessarily bounded net sentiment between −100% and +100%. A neutral category is also constructed, although it is not used in the calculation of net sentiment.

While sentiment was initially measured using the NLP capabilities of the Netbase platform, researchers analyzed initial search results and the keywords driving sentiment associated with media hits, both negative and positive, to determine “contextual correctness” within the subject matter. Within the NetBase platform analysis, the term “weird” has a negative sentiment association by default. Search terms initially returned references to “weird food” and, by default, were classified as having negative sentiment. Contextually, because weird food may elicit a positive sentiment based on novelty and sensationalism in the context of the fair, the NetBase search program was customized to classify “weird” as a neutral term in the analysis.

## RESULTS AND DISCUSSION

Terms associated with agricultural fairs were well represented among social and online media with 2,091,350 mentions during the period of July 1, 2017 12:00 a.m. to September 15, 2019 11:59 p.m. (Table 1). During that time period, 11.5% of those mentions included a reference to food and 12.0% included a reference to livestock. Contrary to the authors’ expectation that agricultural fair social and online media would be dominated by mentions of traditional fair foods, livestock as a topic proved to be similarly popular. Of the fair mentions, including a reference to livestock, 27.5% specifically included dairy- and/or beef-associated terms, 15.8% specifically included poultry-associated terms, and 12.5% specifically included porcine-associated terms. It would thus appear that, among the major food animal livestock species, cattle-related conversation dominated social and online media pertaining to fairs and livestock, while poultry and swine commanded smaller but detectable portions.

**Table 1.** Number of mentions and sentiment associated with agricultural fair conversations and fair conversations, including references to livestock, cattle (beef and dairy), poultry, swine, and food during the time period July 1, 2017 through September 15, 2019

Fair topic narrowed by theme	Agricultural Fair Topic					
	All fair conversation	Livestock	Beef and dairy	Poultry	Swine	Food
Total number of mentions	2,091,350	250,750	68,900	39,600	31,250	239,800
w/ sentiment	186,900	19,200	5,600	4,100	2,400	21,300
w/ an attribute	117,150	13,200	4,250	2,850	2,200	11,150
Positive sentiment	78%	94%	98%	95%	90%	98%
Negative sentiment	22%	6%	2%	5%	10%	2%
Net sentiment	56%	88%	96%	90%	79%	96%

Net sentiment is quantified as the total percentage of positive posts minus the percentage of negative posts, which results in a necessarily bounded net sentiment between −100% and +100%.

**Figure 2.**
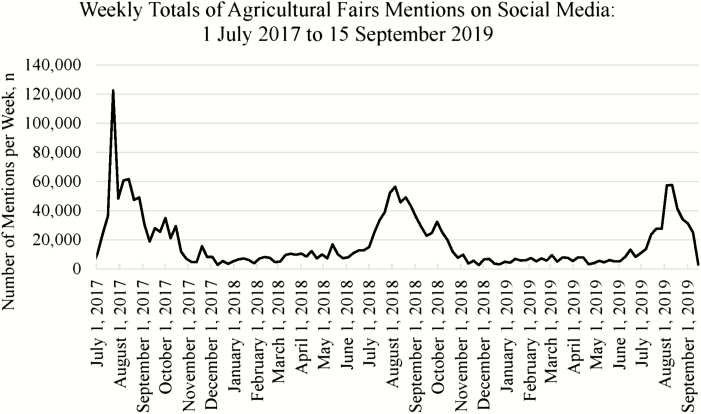
The number of social media mentions containing agricultural fair query keywords was cyclical with most chatter occurring during the months of July, August, and September each year.

Social and online media concerning agricultural fairs was clearly cyclical. The majority of media generation occurred during the summer months of July, August, and September each year as shown in Fig. 2. The greatest volume of chatter occurred during the month of August, while the period of November to June was characterized by minimal amounts of fair-related media. Indeed, the majority of fairs in the United States occur during the months of July through October (Lillywhite et al., 2013b) and the majority of state fairs occur in August (35%), September (27%), and October (23%; Supplementary Data). Over the 27-mo period of interest, the greatest number of fair-related mentions occurred during the week of July 23, 2017, which corresponds to the occurrence of a fatal carnival ride accident at the Ohio State Fair on July 26, 2017 (Almasy and Chavez 2017; Helsel 2017). This accident resulted in the death of one person and the hospitalization of seven additional persons and would appear to have made the largest impact on fair-related social media conversation across the three fair season time period analyzed. During this period of social listening, variant influenza A (H3N2) infection occurred in 40 Maryland agricultural fair attendees resulting in the hospitalization of two children in September 2017 (Duwell et al., 2018) and, in 2018, the first variant viral infection (influenza A(H3N2) variant) was reported in July as having occurred in a youth attendee of an Indiana agricultural fair (CDC, 2018). In comparison to the carnival ride incident, these events did not dominate online media in our search.

**Figure 3.**
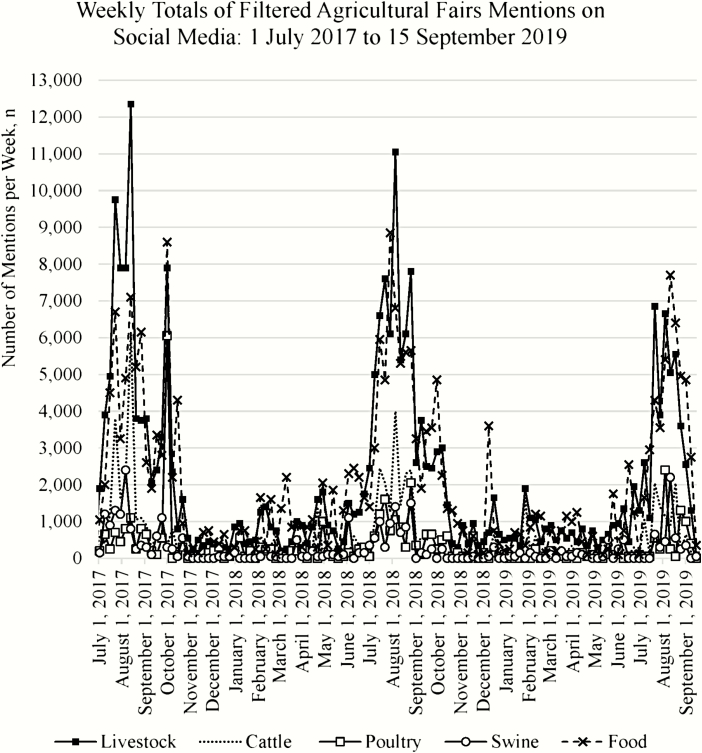
The number of social media mentions about agricultural fairs, which also contained livestock-, species- (cattle, poultry, or swine), or food-specific keywords, was cyclical and erratic over time for individual topic categories.

When fair-related online media was categorized as referencing food, livestock, cattle, poultry, or swine, similar patterns of cyclicity were evident (Fig. 3). Conversations relating to the fair and either food, livestock, cattle (beef or dairy), poultry, or swine were highest during the summer months but waned in the fall and remained low throughout the winter, spring, and early summer seasons. Although the total number of fair mentions referencing livestock actually surpassed the total number of fair mentions referencing food over the 27-mo time period (Table 1), on a weekly basis, the volume of livestock search results was not consistently greater than results about food (Fig. 3). Further analysis showed that, on weekly intervals, the percentage of fair-related mentions that referenced food ranged from as low as 0.9% up to 51.4%. Similarly, the weekly percentage of fair-related mentions that referenced livestock ranged widely from 2.7% to 44.6% of the fair-related conversation. Therefore, a considerable portion of agricultural fair search results in online and social media might include livestock references depending on the specific time interval investigated.

Among the food animal livestock subsets, cattle received the most mentions (*n* = 68,900), thereby exceeding the total mentions of poultry (*n* = 39,600), while swine commanded less than half of the mentions of beef and dairy (*n* = 31,250; Table 1). On weekly intervals throughout the 27-mo period (Fig. 3), it is interesting to note the variability in species-specific chatter; of the livestock-focused mentions, the volume of search results specifically referencing cattle ranged from 0% to 86.8%, while chatter specifically referencing poultry (0–85.7%) or swine (0–76.9%) had similar percentage ranges. Thus, even though swine did not consistently garner a large proportion of attention in social and online media in the context of agricultural fair livestock, the potential to dominate the conversation during certain time periods is evident.

The number of fair-related posts is necessarily less than the number of fair-related mentions because multiple mentions (sentences) can be contained within a single post (NetBase, 2018). The volume of fair-related posts mirrored the percentages of mentions with the most posts occurring during the summer months and peaking in August each year. The volume of fair-related posts that also included references to food, livestock, and specifically to cattle, poultry, or swine, similarly was greater in the summer months and was minimal throughout the months of November to June, mirroring the pattern observed for mentions (Supplementary Data). While the number of posts referencing food and livestock had strong unimodal peaks in August 2018 and 2019, there was bimodal peak for the number of food and livestock posts in 2017 during the months of August and October 2017.

Of the portion of posts with a known day of the week (*n* = 313,500) and time of the day (*n* = 17,000) for the respective post, posts referencing livestock were relatively low on Sunday and Monday compared to the other days of the week (Table 2). Demographic information and self-described interests were available for a portion of the authors of fair-related posts and fair-related posts that also specifically referenced livestock (Table 3). Among the fair-related posts associated with an author gender (*n* = 666,900), a relatively close split existed between male (48%) and female (52%) posters. However, fair-related posts that specifically referenced livestock and had a known author gender (*n* = 64,000) were published more frequently by females (55%) than by males (45%). Fair-related posts that were associated with an inferred author age (*n* = 679,323) were greatest among the inferred age groups of 25–34 and 55–64 yr. Fair-related posts were relatively sparse among posters younger than 25 and older than 64 yr of age. A similar age distribution among authors was evident for posts related to a fair and referencing livestock by posters having an inferred age (*n* = 64,635). On a national scale, Pew Research Center data historically shows that the age distribution of social media users is greatest among young adults and progressively lessens with increasing age brackets (Greenwood et al., 2016; Smith and Anderson 2018; Perrin and Anderson 2019). In comparison, the present data shows a notable proportion of social media fair discussion among the 55–64-yr-old age group. Further research would be helpful to determine if there is disproportionate social media activity among this age group associated with higher fair attendance and/or other generational considerations, such as being a grandparent.

**Table 2.** Timing associated with authors engaging in social media conversations related to agricultural fairs and livestock during the time period July 1, 2017 through September 15, 2019

	Fair-related posts	Fair-related posts that reference livestock
Posts by day of week (*n*)	313,500	17,000
Monday	13%	9%
Tuesday	11%	18%
Wednesday	17%	21%
Thursday	18%	15%
Friday	17%	18%
Saturday	14%	12%
Sunday	11%	9%
Mentions (*n*)	2,091,350	250,750
All Posts (*n*)	1,732,900	182,450

**Table 3.** Demographics and top self-described interests associated with authors engaging in social media conversations related to agricultural fairs and livestock (July 1, 2017 to September 15, 2019)

Fair-related posts		Fair-related posts filtered by livestock	
Gender (*n*)	666,900		64,000
Male	48%		45%
Female	52%		55%
Inferred Age (*n*)	679,323		64,635
<18	10%		7%
18–24	13%		11%
25–34	18%		18%
35–44	15%		17%
45–54	15%		16%
55–64	18%		19%
65+	11%		12%
Posts by authors with self- described interests	*n* = 353,500		*n* = 24,500
Top five interests	% of posts	Top five interests	% of posts
Family	31%	Family	24%
Politics	17%	Music	14%
Music	16%	Politics	12%
Food and Drink	13%	Food and drink	12%
Religion	13%	Photo and video	10%
Mentions (*n*)	2,091,350	250,750	
All posts (*n*)	1,732,900	182,450	

Family was the top self-described interest by posters of both fair-related posts (*n* = 353,500) and of fair-related posts referencing livestock. This observation reaffirms the importance of taking precautions to safeguard against zoonotic disease transfer to children. Since the transmission of major zoonotic diseases of concern occurs via oral ingestion (LeJeune and Davis 2004), young children constitute a high-risk category because of their high likelihood of hand-to-mouth activity following livestock exposure in addition to their developing immunocompetence.

Politics, music, and food and drink also surfaced as top interests among posters. It remains indeterminable whether the strong interests in politics and music reflect the naturally associated interests of fair enthusiasts or the extent to which fair programming, that is, hosting political functions and concerts, influences social media posts. Live music has been a mainstay attraction at agricultural fairs throughout their history (Talusan 2004; Marsden 2010; Lillywhite et al., 2013b). Investigation of the online and social media surrounding fairs in the present study revealed frequent reference to political candidates and political appearances in conjunction with fairgrounds or a fair event. The high interest in politics among posters engaging in fair-related online and social media is not surprising considering the desire to foster the appearance of accessibility, build rapport, and generate favorable press, which fairs and fairgrounds offer to campaigning politicians (The Economist 2015; Wood 2016). Further research would be edifying for the agricultural industry to identify political and entertainment-based opportunities for consumer engagement at fair venues.

The majority of livestock fair online and social media was generated by news sources rather than from Twitter in contrast to the distribution of content among sources of other conversation topics and unfiltered fair conversations (Table 4). Therefore, it could behoove the agricultural industry to develop a working relationship with news outlets and establish rapport with reporters as the predominant social media source covering agricultural fairs. Various news sources represented the other top domains for the different (filtered) fair conversations. An agriculturally focused news domain, morningagclips.com, contributed notably to fair conversation, including conversations referencing food that are not necessarily livestock based. For fair conversations referencing poultry, another agriculturally focused domain, backyardchickens.com, was the domain offering the sixth-highest number of mentions.

**Table 4.** Top sources and domains for social media conversations mentioning agricultural fair topics (July 1, 2017 to September 15, 2019)

Topic	Sources, *n*	Source name	% of mentions	Top 10 domains, *n*	Domain address	% of mentions
Agricultural fair	2,091,350	Twitter	62.3%	1,373,050	twitter.com	94.9%
		News	27.6%		reddit.com	2.1%
		Blogs	6.0%		morningagclips.com	0.9%
		Forums	4.0%		fortmorgantimes.com	0.4%
		Customer reviews	0.1%		crestonnews.com	0.3%
		Comments	<0.1%			
Fair and livestock	250,750	News	49.8%	106,200	twitter.com	82.4%
		Twitter	34.9%		morningagclips.com	4.8%
		Blogs	11.3%		reddit.com	3.0%
		Forums	3.9%		fortmorgantimes.com	2.5%
		Customer reviews	0.1%		desmoinesregister.com	1.6%
		Comments	<0.1%			
Fair and cattle (beef and dairy)				28,050	twitter.com	71.3%
					morningagclips.com	6.4%
					fortmorgantimes.com	4.5%
					reddit.com	3.2%
					sfgate.com	3.2%
Fair and poultry				23,600	twitter.com	80.5%
					brainerddispatch.com	4.4%
					thenewstribune.com	2.8%
					reddit.com	2.3%
					chillicothegazette.com	1.9%
Fair and swine				15,250	twitter.com	72.1%
					desmoinesregister.com	8.5%
					morningagclips.com	3.9%
					theolympian.com	3.6%
					chicagotribune.com	2.3%
Fair and food				166,750	twitter.com	93.6%
					reddit.com	1.7%
					morningagclips.com	0.7%
					desmoinesregister.com	0.7%
					mprnews.org	0.6%

### Social Media Sentiment Analysis for Fairs and Subsearches Conducted

**Figure 4.**
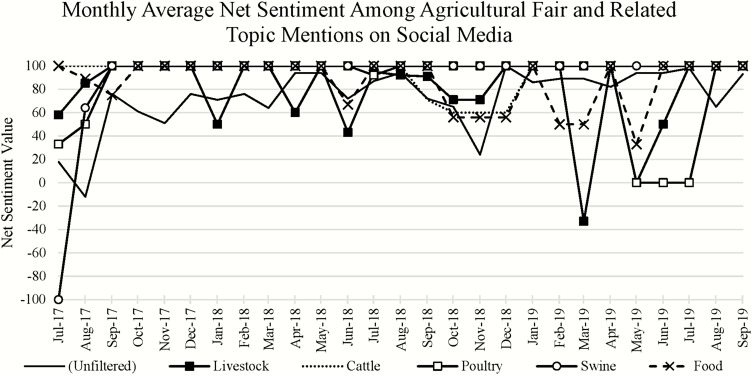
The monthly average net sentiment associated with social media conversations regarding agricultural fairs and filtered fair conversations, which also referenced food, livestock, or species (cattle, poultry, or swine), was predominantly positive over the period of analysis (July 1, 2017 to September 15, 2019) and showed occasional negative net sentiment for certain topics.

Overall, net sentiment associated with mentions of agricultural fairs was 56% on a range of −100 to 100% (Table 1). Mentions specifically related to food, livestock, and particular food animal species yielded even more positive net sentiment. Positive sentiment was highest (98%) for food or cattle-related mentions and was lowest, albeit still wholly positive (90%), for swine-related mentions. Notable fluctuations in net sentiment are evident for the subsearches devoted to livestock (March 2019), poultry (May and June 2019), and swine (July 2017; Fig. 4).

The top sentiment drivers for agricultural fairs are shown in Table 5. Ironically, a large portion of the social media chatter concerning a “horrific accident” was actually in reference to a comical spoof video (in which a fair food item was dropped on the ground), which was categorized by NetBase as negative due to the vernacular referencing the video. Legitimate negative sentiment was driven by negative press concerning the July 2017 fatal carnival ride malfunction.

**Table 5.** Top sentiment driver attributes in agricultural fair conversations on social media (July 1, 2017 to September 15, 2019)

Likes	Dislikes		
Fair	12%	Horrific accident	18%
Great	6%	Reported ride malfunction	2%
Great time	2%	Kill one	0.5%
Festival favorite	1%	County fair employee	0.5%
Day	1%	Ride	0.5%
Total mentions		2,091,350	
Total mentions with sentiment		186,900	
Total mentions with an attribute		117,150	
Net sentiment		56% (78% positive, 22% negative)	

The top five hashtags used in positive conversations associated with agricultural fairs were #decisionamerica, #wednesdaywisdom, #tbt, #atthefair, and #mfucoffeeshop. The #throwbackday and #atthefair hashtags should be considered candidate keywords for future social media monitoring purposes. The top five hashtags used in negative conversations associated with agricultural fairs were: #flipthe7th (a politically affiliated hashtag at the Chesterfield County Fair), #abigailspanberger (referencing a political issue at the Chesterfield County Fair in which a Democratic candidate was video recorded not shaking hands with a woman), #ncf17 (referencing Neshoba County Fair 17, in which there were negative references about political items), #ohio, and #killed (referencing an accident with a ride at a fair).

The top five hashtags included in positive conversations associated with livestock and agricultural fairs were #reservegrandchampion, #4h, #histsci (a hashtag for historical photos from the fair), and #naturalhistory. Thus, the data would suggest that youth livestock exhibition and 4-H programs help to create an opportunity for positive social media attention to livestock at agricultural fairs. A consumer preference study conducted by Lillywhite et al. (2013a) documented strong public utility of animal interaction at fairs on par with that of the carnival amusement ride attractions. Notably, none of the top hashtags among search results were associated with negative conversations concerning livestock and agricultural fairs.

The top sentiment drivers for agricultural fairs focused on livestock-related online and social media search results are shown in Table 6. Unsurprisingly, “adorable baby farm animal” and “rooster” are livestock-specific terms driving positive sentiment. Whilst considerable angst within the livestock industry is derived from consumers’ potentially negative perceptions of animal agriculture, this comprehensive snapshot and analysis of social online media fails to provide evidence of widespread public concern regarding animal handling and/or exhibition. Moreover, sentiment results showed positive sentiment attached to viral posts and media of youth with their livestock, including a particularly popular post of a cow and child napping together. Agricultural industry support of youth via awarding of scholarships also surfaced as a source of positivity associated with swine. The phrase “move in veteran,” which appears as a top sentiment driver, originated from a news outlet that compressed multiple top news stories into a single tweet. Due to the short nature of tweets, news outlets will often compress the information from top news stories into one tweet. For example, information regarding the fair may be included in the same tweet as news about veterans and politics. On occasion, this may result in associated verbiage that is not directly related to the topic being studied. However, removing that information could result in loss of data.

**Table 6.** Top five sentiment driver attributes in agricultural fair conversations on social media that reference livestock species and fair food (July 1, 2017 to September 15, 2019)

Conversation topic	Likes		Dislikes	
Livestock	Total mentions with an attribute: 13,200			
	Fair	11%	Hog barn	0.8%
	Adorable baby farm animal	5%	Shut down	0.8%
	Great	5%	Hog	0.4%
	Move in veteran	4%	Not kick off	0.4%
	Win internet	4%	Lineup	0.4%
Cattle	Total mentions with an attribute: 4,250			
	Fair	15%	Two error	1%
	Fun playing game	12%	Shares	1%
	Hand scooped	12%	—	
	Win internet	12%	—	
	Great	7%	—	
Poultry	Total mentions with an attribute: 2,850			
	Move in veteran	18%	Lineup	2%
	Fun playing game	18%	Rank	2%
	Awesome day	18%	Hog barn	2%
	Corn dog	4%	Shut down	2%
	Reward state’s vociferous rooster	4%	Disaster	2%
Swine	Total mentions with an attribute: 2,200			
	Fair	27%	Hog barn	5%
	Fun playing game	23%	Shut down	5%
	Great	5%	Hog	2%
	Award scholarship	5%	Test for influenza	2%
	Look forward to	2%	—	
Food	Total mentions with an attribute: 11,150			
	Fair	14%	Perfect excuse to go	1%
	Festival Favorite	10%	Soggy start	1%
	Food item	5%	Bust	0.4%
	Move in veteran	4%	Fun	0.4%
	Sweet victory	4%	—	

Terms driving negative sentiment were largely reflective of conversation concerning swine influenza, hog barns, and biosecurity shutdowns. Swine influenza zoonotic concern organically surfaced in fair livestock conversation without probing search terms. Interestingly, because of conversation in which both pigs and chickens were referenced, negative swine influenza online media caused a decrease in the net sentiment measured for poultry simply by association. This finding highlights the value of unified efforts and messaging across food animal species not only due to commonalities of biological risks inherent to livestock exhibition regardless of species but also due to the potential for misdirected negative public sentiment.

State and county fair exhibits have a documented history of usage for public health education and have served as platforms for the introduction of public health education initiatives (Winslow 1916; Harty 1993; Holt 2005). Avian and swine influenza outbreaks highlight the importance of herd health management, biosecurity, and biosafety measures, reasons for which livestock exhibitions and shows have been canceled in the past (DeVita and Reimink 2015; NPPC 2019). Yet, the potential for media coverage to reinforce fears that animal agriculture could jeopardize public health should not be underestimated. Influenza outbreaks among fair animals might elicit public memories of avian influenza and the so-called “Swine Flu” pandemic (Mostafa et al., 2018) and foster unfounded doubts of meat safety and appropriateness of contemporary rearing practices (Maes et al., 2019). Currently, the extent to which the social media coverage of swine flu itself directs public attention to the possibility of zoonotic disease transfer is unclear.

Novel (animal origin) influenza A virus infection in humans, a nationally notifiable condition in the United States, is relatively rare (CDC 2019). For example, during the 3-yr period from 2017 to 2019, total reported cases for H1N1v, H1N2v, and H3N2v were only 2, 17, and 63 cases, respectively (FluView Interactive 2020). However, viral transmission of influenza A from poultry or swine to humans can be unpredictable with potentially severe public health impacts (Dawood et al., 2012; Widdowson et al., 2017). Admittedly, animal to human transmission is not necessarily associated with the duration or intensity of animal exposure, nor is it limited to livestock species since transmission can also occur via wildlife or domesticated felines (Widdowson et al., 2017; Borkenhagen et al., 2019). Consequently, continued research and education are warranted to minimize public zoonotic disease risk factors and influenza exposure, that is, through conjunctival and mucosal membrane contact (Mostafa et al., 2018) at agricultural fairs. Livestock exhibits at fairs should be investigated as a means by which zoonotic disease risk education and public health knowledge may be increased.

Table 7 displays the top sentiment terms in both positive and negative mentions about fairs generally, as well as each of the livestock subsearches and the fair foods subsearch. Interestingly, the only specific fair to show up in the top five positive mentions of the unfiltered search was the New York State Fair. The Iowa State Fair appeared in the top five positive terms within the swine subsearch, which is perhaps unsurprising given the predominance of the swine industry in Iowa (USDA—NAAS, 2019). The Minnesota State Fair appeared as a top five positive term for both poultry and fair foods, which is perhaps unsurprising given the fame attributed to the fair foods there. Top negative terms surrounded accidents and illnesses, among other comments that varied depending on the specific subsearch being studied. Shows appeared commonly as both a positive and a negative term, indicating comments that were both positive (i.e., good show!) and negative in nature about shows.

**Table 7.** Most prevalent sentimental terms included in positive and negative mentions about agricultural fairs and referencing livestock and/or food (July 1, 2017 to September 15, 2019)

Topic:	Positive mentions			Negative mentions		
	*n*	% of positive mentions including top term		*n*	% of negative mentions including top term	
Fairs (unfiltered)	145,750	Love	9%	41,500	Iowa State Fair	8%
		Out	7%		Horrific accident	8%
		Fun	7%		Ohio State Fair	6%
		New York State Fair	5%		@FreeMemesKids	4%
		Show	5%		Ride	4%
Livestock	23,050	Animals	12%	2,400	Shows	8%
		Chicken	9%		Hogs	8%
		Shows	9%		Swine flu	6%
		Ride	9%		Hog barn	4%
		Enjoy	5%		Barn	4%
Cattle	12,400	Cows	15%	350	Show	14%
		Wins	5%		Better	14%
		Animals	5%		Central States Fair pen	14%
		Riding	4%		Cheese curds	14%
		Visiting	4%		Errors	14%
Swine	7,900	Pigs	15%	1,950	Hogs	10%
		Iowa State Fair	8%		Swine flu	8%
		Animals	8%		Swine	8%
		Rides	7%		Hog barn	5%
		Visit	7%		Clinton County Fair	5%
Poultry	14,900	Chicken	14%	1,250	Find	4%
		Rides	7%		Swine flu	4%
		Animals	4%		MAS	4%
		Minnesota State Fair	4%		Out	4%
		visiting	4%		UPDATE	4%
Food	42,850	Foods	20%	1,300	Cotton candy	8%
		Ride	7%		Find	4%
		Minnesota State Fair	7%		Wednesday, Sept.	4%
		August	6%		MAS	4%
		Fun	5%		Soggy start	4%

## CONCLUSIONS AND IMPLICATIONS

Collectively conversations related to agricultural fairs investigated were overwhelmingly positive. Furthermore, these conversations reflect public sentiment that can be very volatile and/or divergent from those in the agricultural community. Posts including references to animals appear to promulgate this positive sentiment, while comments conveying negative sentiment related to impassioned issues, such as slaughter or compromised animal welfare, were minimal to nonexistent. Few legitimate negative concerns arose during this time period but those that did were concerned with compromised human and animal health. The livestock industry cannot ignore the risks and concerns associated with zoonotic disease spread in association with agricultural fairs. The issue organically surfaces within social media conversation and small, regional outbreaks can rapidly saturate widespread social media news. Further investment by the livestock industry into matters concerning animal health management, public health and education, and positive agricultural perception is warranted. This research also suggests the positive potential of developing affable cooperative relationships with news sources and reporters considering their domination of the online discussion pertaining to agricultural fairs.

## Supplementary Material

txaa139_suppl_Supplementary_Material
