# Supplementary material for: #GoingtotheFair: a social media listening analysis of agricultural fairs
Source: Transl Anim Sci. 2020 Jul 20;4(3):txaa139. doi: 10.1093/tas/txaa139 (PMC7433905; doi:10.1093/tas/txaa139)
Supplement: txaa139_suppl_Supplementary_Material [file txaa139_suppl_supplementary_material.docx]

**Electronic Supplements (E-Supplements)**

**Appendix A. Search Terms for the Primary Search of “Agricultural Fairs.”**

*Primary Terms:* agricultural fair, #agriculturalfair, #agfair, state fair, #statefair, county fair, #countyfair, parish fair, youth fair, #youthfair, going to the fair, #goingtothefair

*Exclude Terms:* free & fair election; free, FAIR elections; free, fair and credible; free, fair, and open internet; fair trade

*Neutral Insights:* weird

**Appendix B. Search Terms Filtering the Primary Search Related to “Agricultural Fairs” to Document Media Hits Specific to Food.**

*Date Range:* 1 July 2017 12:00 AM to 15 September 2019 11:59 PM

*Include Terms:* fair food, #fairfood, hungry, #hungry, lemonade, #lemonade, eat, #eat, fried, popcorn, #popcorn, cotton candy, #cottoncandy, caramel apple, #caramelapple, oreo, funnel cake, #funnelcake, elephant ear, #elephantear, treat, cream puff, #creampuff, caramel corn, corn, cookie, fry bread, doughnut, pickle, nuts, taco, potato, potatoes, pie, nachos, pizza, fries, fritter, root beer, #caramelcorn, #frybread, #doughnut, #pickle, #nuts, #taco, #potato, #pie, #nachos, #pizza, #fried, #fries, #fritter, #rootbeer, #gyro, gyro, chocolate, #chocolate, pork, #pork, burger, #burger, corn dog, #corndog, pork chop, #porkchop, loin, #loin, sausage, #sausage, bacon, #bacon, hot dog, #hotdog, ribs, #ribs, BBQ, #bbq, barbeque, #barbeque, barbecue, #barbecue, egg, turkey, turkey leg, #egg, #turkeyleg, chicken, #chicken, drumstick, #drumstick, milk, #milk, ice cream, #icecream, cheese, #cheese, curds, #curds, cheese bites, #cheesebites, milkshake, #milkshake, beef, #beef, hamburger, #hamburger, steak, #steak, bratwurst, #bratwurst, veal, #veal, lard, #lard, #chocolatemilk, #cheesecurds, beefitswhatsfordinner, #beefitswhatsfordinner, pulled pork, #pulledpork, #foodpics, #porkribs, #wholehog, #porkbelly, food, #food, #yummy, #delicious, #lunch, #dinner, #foodporn, #foodphotography, #foodlover, #foodgasm, #foodie, #foodstagram, #tasty, ribeye, butter, tenderloin, #theotherwhitemeat, #porkbeinspired, #beinspired, itswhatsfordinner, #delish, #foodintheair, #eatingfortheinsta, #foodpornography, #nom, #InstaGood, #InstaFood, #Food, #IGEats, #Foodstagram, #ChefMode, #FoodSpotting, #CleanEating, #meat, #grill, #grilling, #meatlover, #foodblogger, #instafood, #instagood, #Foodie, #yum, #cleaneating, #foodspotting, #igeats, #chefmode, #ilovesharingfood, #iatethis, #IAteThis, #breakfast, #brunch, #snack, #meal, #supper, #corndogs, #porkloin, pork loin, #ilovebacon, #hotdogs, pork burger, #porkburger, #porkburgers, bbq, #porkbbq, #porkbarbecue, #porkbarbeque, #porkrib, #eggs, #turkeylegs, #drumsticks, #chickenwing, #chickenwings, #wings, #hamburgers, #steaks, sausage, #beefbbq, #beefbarbecue, #beefbarbeque, #itswhatsfordinner, #beefribs, #beefsausage, dairy, #dairy, #icecreamcone, #strawberrymilk, #milkshakes, #caramelapples, #funnelcakes, #elephantears, #creampuffs, #doughnuts, #pickles, #tacoes, #potatoes, #tacos, #pies, #fritters, #gyros, #burgers, #porkchops, #sausages, #dumsticks, #icecreamcones, #bratwursts, #butter

**Appendix C. Search Terms Filtering the Primary Search Related to “Agricultural Fairs” to Document Media Hits Specific to Livestock.**

*Date Range:* 1 July 2017 12:00 AM to 15 September 2019 11:59 PM

*Include Terms:* pig, cow, barn, chicken, livestock, #livestock, sow, #sow, swine, #swine, #pig, piglet, #piglet, porcine, #porcine, sheep, #sheep, lamb, #lamb, beef, dairy, #beef, #dairy, cattle, #cattle, #cow, bull, #bull, calf, #calf, calves, #calves, steer, #steer, heifer, #heifer, purebred, breed, goat, #goat, duck, #duck, #ducklings, turkey, #turkeys, hen, #hen, rooster, #rooster, #chicken, chick, #chick, goose, #goose, geese, #geese, gosling, #gosling, rabbit, #rabbit, bunny, #bunny, bunnies, #bunnies, horse, #horse, pony, #pony, ponies, #ponies, animal, #animal, pet, petting, #petting, #barn, rodeo, #rodeo, poultry, #poultry, crow, #crow, tail, feather, egg, #egg, avian, #avian, #bovine, bovine, pen, #pen, cage, farrow, #farrow, gilt, #gilt, barrow, #barrow, boar, #boar, calve, #calve, calving, #calving, #feather, crowing, #crowing, gobble, #gobble, oink, #oink, moo, #moo, #pigs, #cows, #barns, #chickens, #sows, #piglets, #goats, #ducks, #duckling, #turkey, #hens, #roosters, #chicks, #goslings, #rabbits, #horses, #animals, #rodeos, #feathers, #eggs, #cage, #boars, manure, #manure, #babypig, #babysheep, #babyhorse, #babyduck, #babychicken, #babycow, #babygoat, #babyturkey, #babypigs, #babyhorses, #babysheeps, #babyducks, #babychick, #babychickens, #babychicks, #babycows, #babygoats, #babyturkeys, #babybunnies, #babyrabbits, #babybunny, #babyrabbit, #poultryofig, #poultryofinstagram, #hensofinstagram, #hensofig, #chickensofig, #chickensofinstagram, #pigsofinstagram, #pigsofig, #cowsofig, #cowsofinstagram, #calfs, #cowbarn, #pigbarn, #swinebarn, #ducksofinstagram, #ducksofig, #roostersofig, alpaca, llama, #alpaca, #llama, #alpacas, #llamas, duckling, #dairybarn, #rabbitsofig, #rabbitsofinstagram, #horsesofig, #horsesofinstagram, #sheepofig, #sheepofinstagram, #goatsofig, #goatsofinstagram, farrowing, udder, milking, #roostersofinstagram, #udder, #udders, #milking, #gilts, #barrows, #chickenbarn, #poultrybarn, #gobbles, #turkeysofig, #turkeysofinstagram, #roostersofinstagram, #dairies, #steers, #heifers, #beefbarn, #bulls, #poniesofinstagram, #poniesofig, #alpacasofig, #alpacasofinstagram, #llamasofig, #llamasofinstagram

**Appendix D. Search Terms Filtering the Primary Search Related to “Agricultural Fairs” to Document Media Hits Specific to Cattle (Beef and Dairy).**

*Date Range:* 1 July 2017 12:00 AM to 15 September 2019 11:59 PM

*Include Terms:* dairy, #dairy, dairy barn, #dairybarn, bovine, #bovine, cow, #cow, cattle, #cattle, udder, #udder, milking, #milking, calf, #calf, calve, #calve, calving, #calving, calves, #calves, #moo, moo, #dairies, #cows, #udders, #calfs, beef, #beef, #beefbarn, bull, #bulls, #bull, steer, heifer, #heifer, #steers, #heifers, #cowsofinstagram, #cowsofig, #cowbarn, #babycow, #babycows

**Appendix E. Search Terms Filtering the Primary Search Related to “Agricultural Fairs” to Document Media Hits Specific to Poultry.**

*Date Range:* 1 July 2017 12:00 AM to 15 September 2019 11:59 PM

*Include Terms:* poultry, #poultry, poultry barn, chicken barn, #chickenbarn, duck, #duck, duckling, #ducklings, turkey, #turkey, gobble, #gobble, hen, #hen, rooster, #rooster, crow, #crow, crowing, #crowing, chicken, #chicken, chick, #chick, goose, #goose, geese, #geese, gosling, #gosling, feather, egg, #egg, avian, #avian, #feather, #poultryofinstagram, #chickensofig, #hensofinstagram, #poultrybarn, #ducks, #duckling, #turkeys, #gobbles, #hens, #roosters, #chickens, #chicks, #goslings, #feathers, #eggs, #poultryofig, #chickensofinstagram, #hensofig, #ducksofinstagram, #ducksofig, #turkeysofig, #turkeysofinstagram, #roostersofig, #roostersofinstagram, #babyduck, #babychicken, #babyturkey, #babyducks, #babychicks, #babychickens, #babyturkeys, #babychick

**Appendix F. Search Terms Filtering the Primary Search Related to “Agricultural Fairs” to Document Media Hits Specific to Swine.**

*Date Range:* 1 July 2017 12:00 AM to 15 September 2019 11:59 PM

*Include Terms:* sow, #sow, swine, #swine, pig, #pig, piglet, #piglet, pig barn, #pigbarn, porcine, #porcine, gilt, #gilt, barrow, #barrow, boar, #boar, farrow, #farrow, oink, #oink, #sows, #pigs, #piglets, #gilts, #barrows, #boars, farrowing, #pigsofig, #pigsofinstagram, #swinebarn, #pigbarns, #babypig, #babypigs
